# Intra- and inter-operator reliability assessment of a novel extramedullary accelerometer-based smart cutting guide for total knee arthroplasty: an in vivo study

**DOI:** 10.1007/s00264-022-05571-2

**Published:** 2022-09-14

**Authors:** Giulio Maria Marcheggiani Muccioli, Domenico Alesi, Arcangelo Russo, Mirco Lo Presti, Iacopo Sassoli, Matteo La Verde, Stefano Zaffagnini

**Affiliations:** 1 II Orthopaedic and Traumatologic Clinic, IRCCS Istituto Ortopedico Rizzoli, Via G.B. Pupilli 1, 40136 Bologna, Italy; 2 University of Bologna, Via Zamboni, 33, 40126 Bologna, Italy; 3 Orthopaedic and Traumatologic Unit, Umberto I Hospital, Azienda Sanitaria Provinciale Di Enna, Enna, Italy

**Keywords:** Smart cutting guide, Inertial sensor, Total knee arthroplasty, Computer-aided surgery

## Abstract

**Purpose:**

The purpose is to verify the intra- and inter-operator reliability of an extramedullary (EM) accelerometer-based smart cutting guide for distal femoral resection during primary total knee arthroplasty (TKA). The hypothesis of the present study was that the use of the device would result in a good correlation between different operators with a difference between repeated measurements of less than 1°.

**Methods:**

Twenty-five not consecutive patients with knee osteoarthritis undergone to primary TKA using an EM inertial-based cutting guide to perform distal femoral resection. In order to assess the agreement in femoral axis definition of the device, two operators performed three time each the manoeuvres necessary to define axis. Inter-rater agreement was evaluated with Bland and Altman agreement test. Intra-rater repeatability was evaluated analysing average results distribution of repeated measurements. Accuracy of the device was evaluated comparing differences between intra-operative device data with final implant alignment measured on post-operative longstanding x-rays using Students’ *t* test.

**Results:**

Agreement between the two operators was statistically significant (*p* < 0.05) with a bias of − 0.4° (95% CI − 0.6° to − 0.2°). Average difference between cut orientation measured with device and final implant position, measured on x-rays, was 0.2° (95% CI − 1.5° to 1.7°) with no statistical difference between the two measurements. Final implant alignment, measured on x-ray, was 90.2°, with 95% of cases distributed within range 88.0° to 92.0° for varus-valgus and 2.8° and with 95% of cases distributed within range 2.0° to 4.0° for flexion–extension.

**Conclusions:**

The EM accelerometer-based smart cutting guide used to perform distal femoral resection during primary TKA demonstrated a good intra- and inter-operator reliability in the present in vivo study.

## Introduction

Coronal and sagittal alignment in total knee arthroplasty (TKA) is essential to achieve a stable implant with long survival, regardless of the type of alignment adopted [1]. Intramedullary reference systems used for femoral may not be accurate [2–5] and, in the presence of extra-articular deformities or hardware in the distal femur, cannot be used. In this case, traditional navigation systems can be used, which require a long learning curve, or, alternatively, navigation systems based on the use of inertial sensors, which have a shorter learning curve but the same accuracy, are becoming more widespread.

The accelerometer-based navigation is simpler to use and less expensive respect to conventional navigation, and it can be used with almost all TKA [6].

However, the reliability of these devices and their superiority to traditional navigation systems has not been widely demonstrated. A recent systematic review analysed the results on the use of inertial sensors, concluding that the benefits on alignment are small while no benefits were found in terms of functional outcome or risk of complications or re-intervention [7]. Otherwise, other authors have demonstrated that these sensors are reliable, comparing in vitro both intra- and inter-operator variabilities but only for tibial resection [8].

The main advantage of inertial sensors over traditional intramedullary instrumentation is the possibility to manage cases with complex extra-articular deformities, obtaining a correct alignment. This technology bases its registration on leg movements. For femoral mechanical axis determination a pivoting movement around femoral head is done to generate angular velocities and accelerations necessary for axis calculation. This registration phase is highly dependent by the operator and by the patient. Its reliability has never been evaluated in surgical setup.

In order to assess the reliability of these inertial sensors, a non-invasive extramedullary (EM) device based on the use of inertial sensors for the positioning of the distal femoral cutting guide has been evaluated.

The aim of the study was to verify the intra- and inter-operator reliability of this EM smart cutting guide on the same patients during primary TKA surgery.

The hypothesis of the present study was the finding of good correlation between operators and a difference < 1°, indicating a good reliability of the device.

## Material and methods

**Fig. 1:**
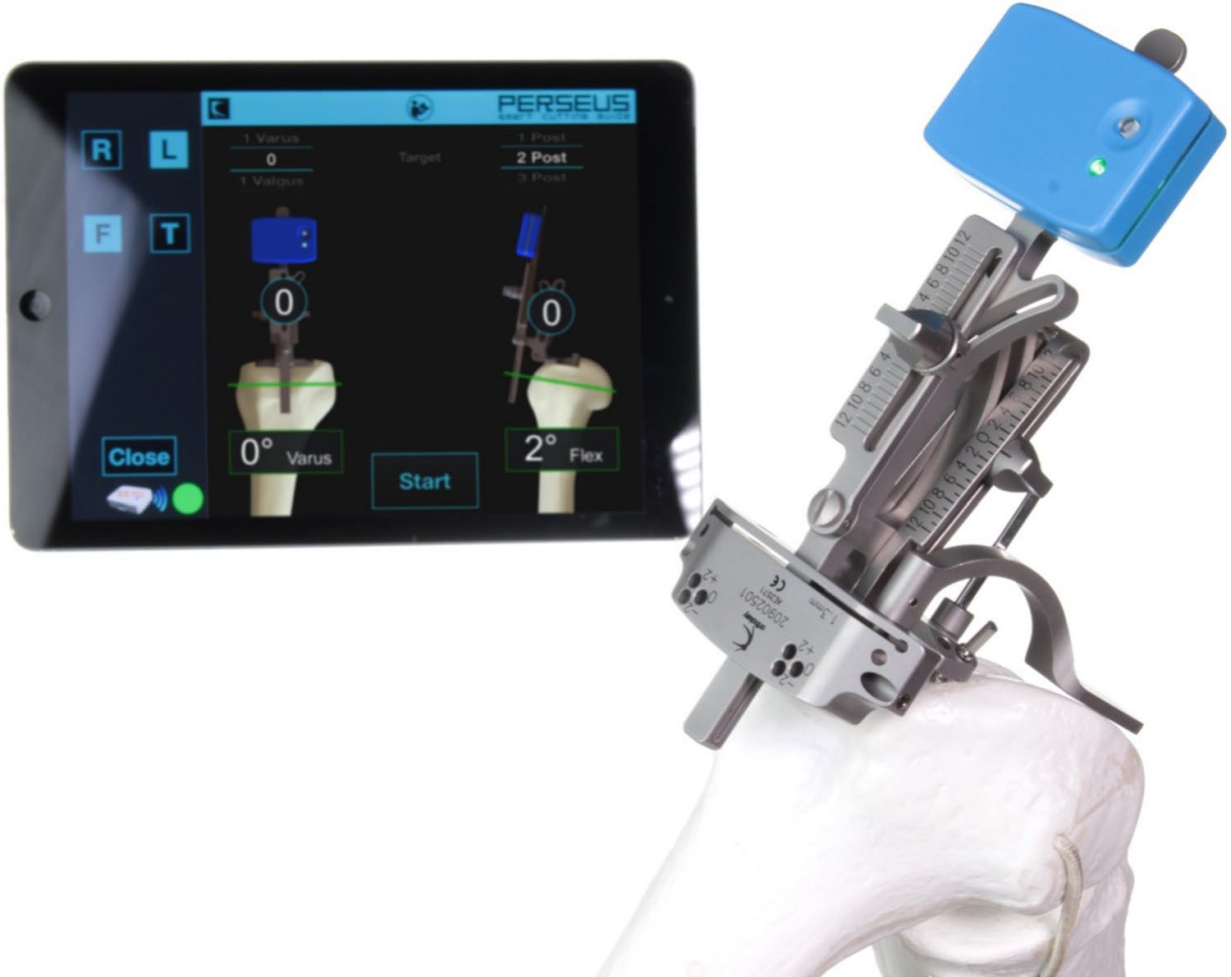
Perseus surgical instrument mounted on femur and interface shown on screen

From January 2019 to October 2021, 25 not consecutive patients with knee osteoarthritis undergone to primary TKA with the aid of an EM inertial-based smart cutting guide to perform femoral resection (Perseus, Orthokey, Italy). The system consists of a cutting guide with disposable devices containing inertial sensors that communicate via Bluetooth with a tablet (Fig. 1). The cutting guide is attached to the distal femur trough a pin positioned at the distal end of the femoral mechanical axis. The system, through the identification of the femoral mechanical axis, directs the surgeon on how to align the cutting guide mounted on the mechanical jig for the resection. Once guide is mounted on distal femur, an initial acquisition is then performed to identify how much to correct the guidance in order to reach the target in varus-valgus (VV) and flexion–extension (FE) established for the distal femoral cut. Correction is then performed by the surgeon, and a second registration is done to verify final guide alignment, before performing resections.

**Fig. 2:**
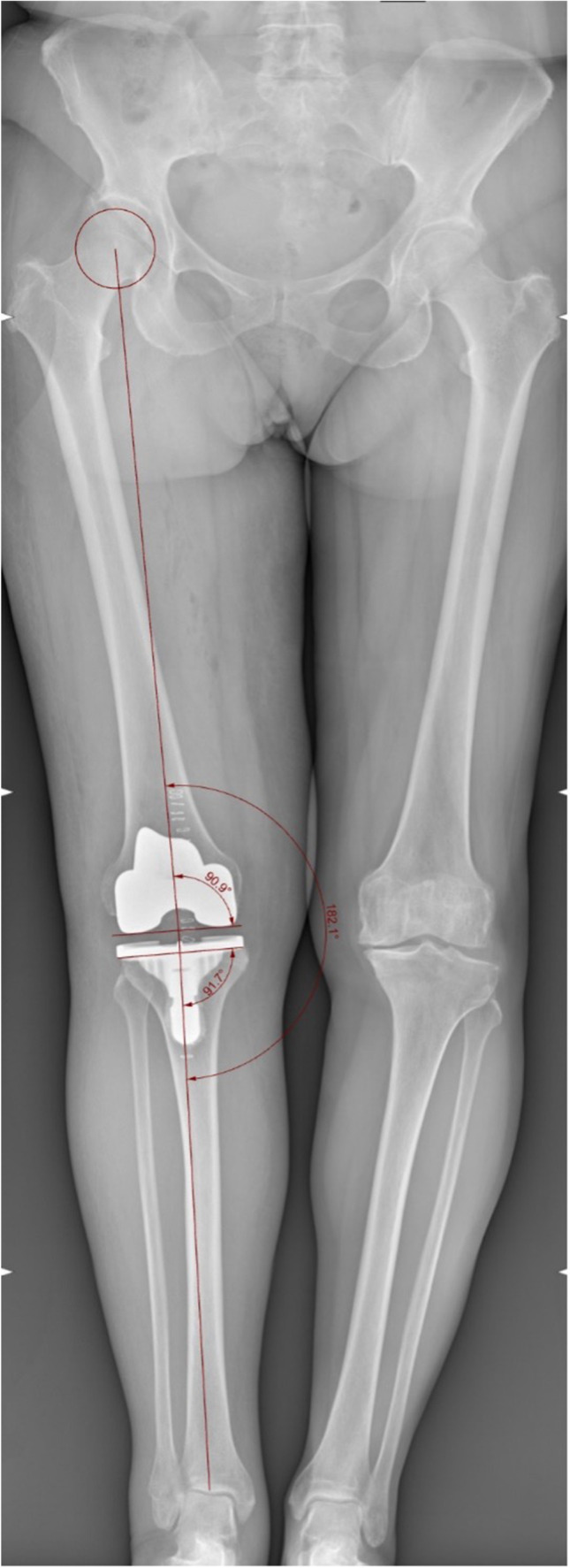
Post-operative x-ray measurement

In order to assess the agreement in femoral axis definition of the device, two operators performed three time each the manoeuvres necessary to define axis. Then, femoral cutting block was oriented, according to surgical plan, with the aid of the device indication; a last acquisition was performed to confirm correct orientation of the cutting jig (a difference with the expected goal of 1° was considered acceptable). Lastly, resection was performed. Inter-rater agreement was evaluated with Bland and Altman agreement test. Intra-rater repeatability was evaluated analysing average results distribution of repeated measurements. Accuracy of the device was evaluated comparing differences between intra-operative device data, on last registration, with final implant alignment measured on post-operative x-rays (Fig. 2) using Students’ *t* test. Measurement on x-ray was done by an independent operator not involved in surgery using Rhinoceros software. Power analysis was based on primary endpoint on agreement between operators. With an alpha of 0.05 and a standard deviation of measurements of 1°, a power of 0.9 can be reached with 25 cases.

Patients were included in this prospective study after approval from the local institutional review board.

The inclusion criteria were patients who were to undergo TKA, with hip mobility range of at least 30°, aged between 40 and 80 years, with a body mass index (BMI) < 35 kg/m^2^.

The exclusion criteria were the presence of ipsilateral hip arthrodesis or ankylosis, a varus or valgus knee deformity > 15° and a BMI > 35 kg/m^2^.

The study cohort consisted of 12 males and 13 females (15 right knees, 10 left knees), with an average age of 68.0 years and a body mass index of 27.9 kg/m^2^.

## Results

**Fig. 3:**
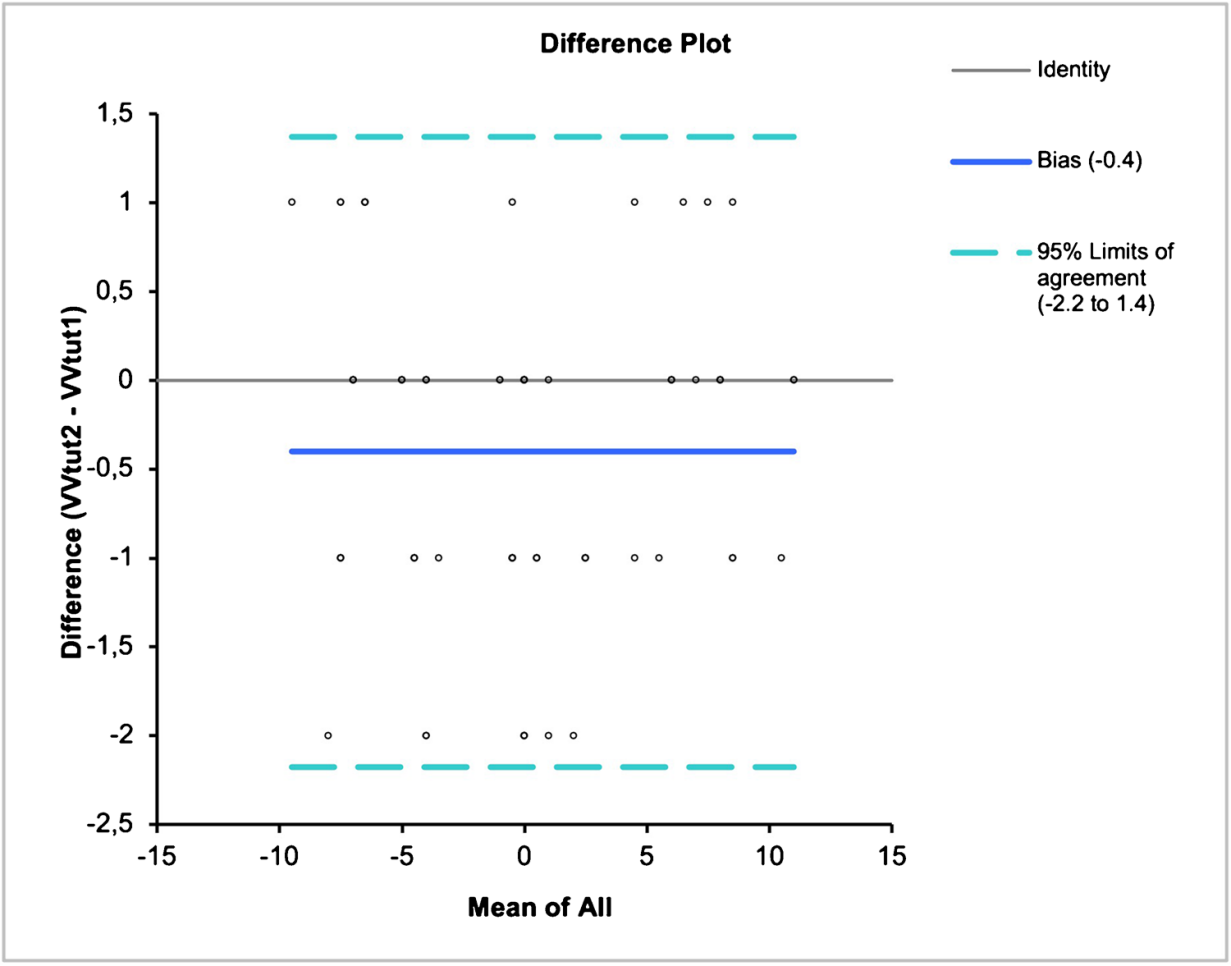
Bland–Altman difference plot

Agreement between the two operators was statistically significant (*p* < 0.05) with a bias of − 0.4° (95% CI − 0.6° to − 0.2°) for varus-valgus orientation and a bias of 0.3° (95% CI 0.1° to 0.6°) for flexion–extension orientation. Data distribution bias and limits of agreement are shown on Fig. 3.

The intra-operator variability is reported as the distribution range of 95% of tests, divided for operator and measured value. Data are reported in Table 1.

**Table 1:** Intra-operator variability reported as the distribution range of 95% of tests, divided for operator and measured value

Measured value	Operator 1		Operator 2	
	95% cases	Range	95% cases	Range
Varus-valgus	0.6°	(0°–2°)	0.8°	(0°–3°)
Flexion	0.7°	(0°–2°)	0.9°	(0°–2°)

### Accuracy

Average difference between cut orientation measured with device and final implant position, measured on x-rays, was 0.2° (95% CI − 1.5° to 1.7°, variance 2.7) with no statistical difference between the two measurements. Final implant alignment, measured on x-ray, was 90.2°, with 95% of cases distributed within range 88.0° to 92.0° for VV and 2.8° and with 95% of cases distributed within range 2.0° to 4.0° for FE.

## Discussion

The main finding of the present study was that both the intra- and inter-operator variabilities of the inertial-based EM cutting guide used to perform femoral resection during primary TKA were within ± 1°, confirming the hypothesis of the study. Using this device in the surgical environment can ensure a good reproducibility between different users and has ensured a final implant orientation within 2° from expected goal.

When compared to computer-assisted surgery (CAS), the versatility of use and quicker surgical time of EM cutting guides provide significant advantages. Navigation systems require a more complex set-up than the EM guide, which only requires attaching the pins to connect the system, with a simple and effective acquisition procedure. According to Goh et al., there was no significant difference in outcomes between the use of EM inertial-based guide and CAS. However, the CAS group’s surgery duration was significantly longer. Moreover, the authors found comparable percentage of patients with an alignment within 3° of a neutral mechanical axis comparing CAS and femoral EM inertial-based system [9].

Bonanzinga et al. found similar results regarding alignment comparing intramedullary and femoral EM inertial-based system [10]. Flexion in the sagittal plane is another important variable in the positioning of the femoral component. In the study of Bonanzinga et al., the EM cutting guide proved better in terms of flexion of the femoral component, but this finding was not significant [10]. Furthermore, a novel study by Bonanzinga et al. compared this device in vitro with CAS in performing tibial cuts, demonstrating comparable accuracy respect to CAS while reducing surgical time and the learning curve and avoiding the risk of pin loosening or infection [8].

The current study had some limitations.

First, the choice of using traditional X-rays in place of computed tomography (CT) implies potential measurement errors due to limb rotation. CT is a more accurate radiologic technique. However, there are several disadvantages with CT, such as metallic artefacts and higher radiation exposure. Additionally, it is challenging to obtain a CT in standing position (a cone beam CT is required to conduct this specific test) [11]. Long-standing hip-to-ankle radiographs can indeed be easily obtained during standard follow-up, avoiding an increase in research costs. Radiographs were repeated if malrotation was detected as radiologists were trained to obtain consistent films. Furthermore, radiographs have been proven to be more radiation-safe and reproducible for determining implant positioning [12, 13]. The second limitation was the limited patient population and lack of a control group. Lastly, it is challenging to draw conclusions about the “learning curve” related with the use of accelerometer-based navigation system from this study.

## Conclusions

The inertial-based EM cutting guide used to perform distal femoral resection during primary TKA demonstrated a good intra- and inter-operator reliability in the present in-vivo study.

## Data Availability

The study data are held by the principal investigator and are available for review.

## References

[1] Lee YS, Howell SM, Won Y-Y (2017). Kinematic alignment is a possible alternative to mechanical alignment in total knee arthroplasty. Knee Surg Sports Traumatol Arthrosc.

[2] Anderson KC, Buehler KC, Markel DC (2005). Computer assisted navigation in total knee arthroplasty: comparison with conventional methods. J Arthroplasty.

[3] Blakeney WG, Khan RJK, Wall SJ (2011). Computer-assisted techniques versus conventional guides for component alignment in total knee arthroplasty: a randomized controlled trial. J Bone Joint Surg Am.

[4] Maderbacher G, Matussek J, Keshmiri A (2018). Rotation of intramedullary alignment rods affects distal femoral cutting plane in total knee arthroplasty. Knee Surg Sports Traumatol Arthrosc.

[5] Maderbacher G, Schaumburger J, Baier C (2016). Appropriate sagittal femoral component alignment cannot be ensured by intramedullary alignment rods. Knee Surg Sports Traumatol Arthrosc.

[6] Batailler C, Swan J, Sappey Marinier E (2020). New technologies in knee arthroplasty: current concepts. J Clin Med.

[7] Budhiparama NC, Lumban-Gaol I, Ifran NN (2019). Does accelerometer-based navigation have any clinical benefit compared with conventional TKA? A systematic review. Clin Orthop.

[8] Bonanzinga T, Giuffrida A, Di Matteo B (2020). In vitro validation of a novel inertial-based cutting guide for tibial resection in total knee arthroplasty. Knee.

[9] Goh GS-H, Liow MHL, Lim WS-R (2016). Accelerometer-based navigation is as accurate as optical computer navigation in restoring the joint line and mechanical axis after total knee arthroplasty: a prospective matched study. J Arthroplasty.

[10] Bonanzinga T, Tanzi P, Pia Neri M (2018). Evaluation of blood loss and implant alignment after total knee arthroplasty with inertial based extramedullary femoral cutting guide. Joints.

[11] Jinzaki M, Yamada Y, Nagura T (2020). Development of upright computed tomography with area detector for whole-body scans: phantom study, efficacy on workflow, effect of gravity on human body, and potential clinical impact. Invest Radiol.

[12] Duparc J, Massare C (1967). Radiological measurement of the angular deviation of the knee in the frontal plane. Ann Radiol (Paris).

[13] Spencer JM, Chauhan SK, Sloan K (2007). Computer navigation versus conventional total knee replacement: no difference in functional results at two years. J Bone Joint Surg Br.

